# Synthetic lethal RNAi screening identifies sensitizing targets for gemcitabine therapy in pancreatic cancer

**DOI:** 10.1186/1479-5876-7-43

**Published:** 2009-06-11

**Authors:** David O Azorsa, Irma M Gonzales, Gargi D Basu, Ashish Choudhary, Shilpi Arora, Kristen M Bisanz, Jeffrey A Kiefer, Meredith C Henderson, Jeffrey M Trent, Daniel D Von Hoff, Spyro Mousses

**Affiliations:** 1 Pharmaceutical Genomics Division, The Translational Genomics Research Institute, Scottsdale, Arizona 85259, USA; 2 Genetic Basis of Human Disease Division, The Translational Genomics Research Institute, Phoenix, Arizona 85004, USA; 3 Clinical Translational Research Division, The Translational Genomics Research Institute, Phoenix, Arizona 85004, USA

## Abstract

**Background:**

Pancreatic cancer retains a poor prognosis among the gastrointestinal cancers. It affects 230,000 individuals worldwide, has a very high mortality rate, and remains one of the most challenging malignancies to treat successfully. Treatment with gemcitabine, the most widely used chemotherapeutic against pancreatic cancer, is not curative and resistance may occur. Combinations of gemcitabine with other chemotherapeutic drugs or biological agents have resulted in limited improvement.

**Methods:**

In order to improve gemcitabine response in pancreatic cancer cells, we utilized a synthetic lethal RNAi screen targeting 572 known kinases to identify genes that when silenced would sensitize pancreatic cancer cells to gemcitabine.

**Results:**

Results from the RNAi screens identified several genes that, when silenced, potentiated the growth inhibitory effects of gemcitabine in pancreatic cancer cells. The greatest potentiation was shown by siRNA targeting checkpoint kinase 1 (CHK1). Validation of the screening results was performed in MIA PaCa-2 and BxPC3 pancreatic cancer cells by examining the dose response of gemcitabine treatment in the presence of either CHK1 or CHK2 siRNA. These results showed a three to ten-fold decrease in the EC_50 _for CHK1 siRNA-treated cells versus control siRNA-treated cells while treatment with CHK2 siRNA resulted in no change compared to controls. CHK1 was further targeted with specific small molecule inhibitors SB 218078 and PD 407824 in combination with gemcitabine. Results showed that treatment of MIA PaCa-2 cells with either of the CHK1 inhibitors SB 218078 or PD 407824 led to sensitization of the pancreatic cancer cells to gemcitabine.

**Conclusion:**

These findings demonstrate the effectiveness of synthetic lethal RNAi screening as a tool for identifying sensitizing targets to chemotherapeutic agents. These results also indicate that CHK1 could serve as a putative therapeutic target for sensitizing pancreatic cancer cells to gemcitabine.

## Background

Pancreatic cancer is one of the most aggressive and lethal cancers known today, with a 5-year survival of only 4%. In 2008, pancreatic cancer was the fourth-leading cause of cancer-related deaths [1]. Patients diagnosed with pancreatic cancer typically have poor prognosis partly because the cancer usually causes no symptoms early on, leading to metastatic disease at the time of diagnosis. The treatment options include chemotherapy, surgery and radiation. The current preferred therapeutic drug to treat pancreatic cancer is gemcitabine, yet the one-year survival of pancreatic cancer patients treated with gemcitabine is only about 18%, representing a significant but modest advancement in the quality of life [2,3].

Gemcitabine (2', 2'-difluoro 2'-deoxycytidine) is a pyrimidine based nucleoside analogue that replaces the nucleic acid cytidine during DNA replication thereby arresting tumor growth since new nucleosides cannot be attached to the faulty nucleoside resulting in apoptosis [4]. Besides pancreatic cancer, gemcitabine is also used for the treatment of various other carcinomas including non-small cell lung cancer [5], ovarian cancer [6] and breast cancer [7]. Due to the poor prognosis of pancreatic cancer, improved therapies are desperately needed and it would be of great benefit to identify agents that sensitize to gemcitabine. Adding other chemotherapeutic agents to gemcitabine has not resulted in meaningful improvement in survival of pancreatic cancer patients. Randomized trials studying the addition of molecular targeting agents (cetuximab, bevacizumab, farnesyl transferase inhibitors and metalloproteinase inhibitors) to gemcitabine compared with gemcitabine alone have been disappointing (for review see [8]). Therefore, newer strategies need to be devised to improve current chemotherapeutic treatments.

In order to identify potential sensitizers to gemcitabine, we employed a functional genomics approach based on high-throughput RNA interference (HT-RNAi) also known as loss-of-function screening. HT-RNAi when combined with drug treatment becomes a platform for identifying synthetic lethality. The basis of this technology is RNA interference (RNAi), a robust method of post-transcriptional silencing of genes using double-stranded RNA (dsRNA) in the form of either siRNA (short interfering RNA) or shRNA (short hairpin RNA) with sequence homology driven specificity [9]. Large-scale libraries of siRNA and shRNA have been used to identify genes involved in many biological functions [10-17]. As kinases are becoming important drug targets for the treatment of cancer, the identification of kinases that act as sensitizing targets to gemcitabine will facilitate the design and development of better drug combinations for treatment of pancreatic cancer.

In this study, our goal was to develop and implement a robust synthetic lethal assay in order to identify genes that potentiate the response to gemcitabine in pancreatic cancer cells. Using a kinase siRNA library, we identified several candidate genes and functionally validated one gene, CHK1, as a sensitizing target using gene specific siRNA in combination with gemcitabine treatment. Furthermore, specific inhibitors of CHK1 were confirmed to have synergistic response with gemcitabine treatment in pancreatic cancer cells.

## Materials and methods

### Cell culture

The human pancreatic cancer cell lines MIA PaCa-2 and BxPC3 were obtained from the American Type Culture Collection (Manassas, VA). The MIA PaCa-2 cell line was established by Yunis, *et al*. in 1975 from tumor tissue of the pancreas obtained from a 65-year-old Caucasian male [18]. The established cell line reportedly has a doubling time of about 40 hours and a colony-forming efficiency in soft agar of approximately 19%. BxPC3 cells were derived from a 61-year-old female with a primary adenocarcinoma of the pancreas. BxPC-3 cells produce mucin and form tumors, which are moderately to poorly differentiated, in nude mice similar to the primary adenocarcinoma. Cells were grown in Dulbecco's modified Eagle medium (DMEM) or RPMI-1640 respectively, supplemented with 10% FBS, 2 mM L-glutamine, 100 IU/ml penicillin G, and 100 μg/ml streptomycin and B. All media reagents were obtained from Invitrogen (Carlsbad, California, USA). The cell lines were routinely maintained at 37°C in a humidified 5% CO_2 _atmosphere.

### Reagents

Gemcitabine chlorohydrate (Eli Lilly; Indianapolis, Indiana, USA) was obtained from the Mayo Clinic Pharmacy (Scottsdale, Arizona, USA) and stock solutions of 100 mM were prepared by dissolving gemcitabine in serum free DMEM. Aliquots of gemcitabine were stored at -20°C until use. The CHK1 inhibitors PD 407824 and SB 218078 were obtained from Tocris (Ellisville, Missouri, USA) and EMD Biosciences (Madison, Wisconsin, USA), respectively and 10 mM stock solutions were prepared in DMSO. Short interfering RNAi targeting CHK1 or CHK2 and a non-silencing control were obtained from Qiagen (Valencia, California, USA). The siRNA target sequences were as follows: CHK1-A, AAGAAAGAGATCTGTATCAAT; CHK1-B, TTGGAATAACTCCACGGGATA; CHK1-C, AACTGAAGAAGCAGTCGCAAGT; CHK1-D, CCCGCACAGGTCTTTCCTTAT; CHK2-A, ACGCCGTCCTTTGAATAACAA; CHK2-B, AGGACTGTCTTATAAAGATTA; CHK2-C, CAGGATGGATTTGCCAATCTT; and CHK2-D, CTCCGTGGTTTGAACACGAAA. The sequences used in HT-RNAi screening were the A and B sequences for both CHK1 and CHK2.

### Synthetic lethal RNAi screening

High-Throughput RNAi (HT-RNAi) was performed using the validated kinase siRNA library version 1.0 obtained from Qiagen. This library includes siRNA to 572 kinases with 2 siRNA per gene that have all been validated by quantitative real time PCR (qRT-PCR) to silence mRNA up to 75%. Stock siRNA was diluted in siRNA buffer (Qiagen) and 9.3 ng of siRNA was printed onto white Corning 384-well plates (Fisher Scientific; Pittsburgh, PA). HT-RNAi was done by reverse transfection of cells. Briefly, diluted siLentFect reagent (BioRad, Hercules, CA) in OptiMEM (Invitrogen) was added to the wells and allowed to complex with siRNA for 30 min at room temperature. MIA PaCa-2 cells were resuspended in growth media without antibiotics at a final concentration of 1000 cells/well. Plates were incubated at 37°C with 5% CO_2_. After 24 hours, either vehicle (serum free media) or gemcitabine was added to the wells and plates were further incubated for 72 hours. The final siRNA concentration is 13 nM. Total cell number was determined by the addition of Cell Titer Glo (Promega, Madison, Wisconsin, USA) and relative luminescence units (RLU) were measured using an EnVision plate reader (Perkin-Elmer, Wellesley, Massachusetts, USA). Raw RLU data was used to calculate viability relative to the control wells. Log_2 _ratios of viability from siRNA and gemcitabine treated wells versus siRNA and vehicle treated wells were computed. Hits were identified as having log_2 _ratios that are 1.65 standard deviations (SD) below the mean ratio level. This cutoff was chosen due to the relatively small size and focused nature of the screen.

### Validation of gene silencing

To demonstrate the silencing efficiency of the siRNA targeting CHK1 or CHK2, MIA PaCa-2 were transfected with 16 nM of siRNA targeting CHK1 or CHK2 or non-silencing siRNA in 6-well plates by reverse transfection as described above. The experiment was run in duplicate and cells were incubated at 37°C for 48 hours prior to RNA extraction or 72 hours prior to preparation of protein lysates for Western Blotting.

### Quantitative real time PCR

RNA extraction was done using Qiagen RNAeasy kit (Qiagen) and cDNA was prepared using iScript cDNA synthesis kit (BioRad Laboratories, CA) [19]. Quantitative real-time PCR using TaqMan assays (Applied Biosystems) was performed to verify gene silencing of CHK1/CHK2 (Hs00967502_m1 and Hs01007290_m1, respectively). The relative quantification was done using the Ct values, determined for triplicate reactions for test and reference samples for each target and for the internal control gene [GAPDH; (Hs99999905_m1)]. Relative expression levels were calculated as 2^-ΔΔCt^, where ΔΔCt = ΔCt (target sample) - ΔCt (reference sample) [19].

### Western blot analysis

Cells were treated with siRNA for 72 hours and cell lysates were prepared as described previously [20]. Protein concentration was determined by BCA assay (Pierce; Rockford, Illinois, USA) and lysates were resolved by SDS-PAGE on 4–12% resolving gel. Proteins were transferred onto PVDF (polyvinylidene fluoride) membranes (Invitrogen) and CHK1 protein was identified using a mouse-anti-CHK1 monoclonal antibody (Santa Cruz Biotechnology; Santa Cruz, California, USA) and an HRP-conjugated goat anti-mouse secondary antibody (Jackson ImmunoResearch Laboratories, Inc; West Grove, Pennsylvania, USA). Bound antibodies were detected using SuperSignal West Femto (Pierce) and imaged using an AlphaInnotech Imager.

### Functional validation for gemcitabine sensitization

For siRNA and gemcitabine studies, cells were transfected with siRNA plated in 384-well plates similar to screening conditions. Twenty-four hours later, the cells were treated with varying doses of gemcitabine in quadruplicate wells for each siRNA plus gemcitabine condition. Cell viability was determined 72 hours after drug addition using Cell Titer Glo. For CHK1 inhibitor studies, cells were treated with either SB 218078 or PD 407824 in 384-well plates for twenty-four hours prior to gemcitabine treatment. Cell viability was determined 72 hours after gemcitabine addition using Cell Titer Glo. Viability was calculated by dividing the average of the RLU values for the drug treated wells by the average of the RLU values for vehicle treated wells. The IC_50 _values were determined using GraphPad Prism (GraphPad Software, San Diego, California, USA) and values were shown as calculated IC_50 _+/- 95% confidence interval.

### Label-free impedance measurement of cell growth

The principle of impedance measurement for monitoring cellular proliferation has been previously described by Solly *et al*. [21]. Briefly, siRNA was introduced into MIA PaCa-2 cells by reverse transfection of 4,000 cells/well using siLentFect in triplicate wells of an ACEA 96× E-Plate (ACEA Biosciences; San Diego, California, USA). Gemcitabine was added at a final concentration of 10 nM at 24 hours after transfection of the cells. The attachment, spreading and proliferation of cells were continually monitored every 60 minutes up to 150 hours, and changes in impedance were acquired with the real time cell electronic sensing (RT-CES) system (ACEA Biosciences). Cell growth was determined by plotting cell index measurements versus time.

## Results

### Synthetic lethal screening for modulators of gemcitabine response

In order to identify genes that modulate the response of pancreatic cancer cells to gemcitabine treatment, we performed synthetic lethal screening using high throughput RNAi. A robust HT-RNAi assay was developed that allowed for high efficiency siRNA transfection of MIA PaCa-2 pancreatic cells by cationic lipids in 384-well plates. Before the actual HT-RNAi screening, a transfection optimization was performed using a panel of commercially available transfection reagents and siLentfect was chosen as it showed the optimal transfection efficiency (Data not shown). We performed a drug dose response experiment with varying concentrations of gemcitabine and chose 5 and 10 nM final concentrations, as we obtained EC_10–30 _doses at these treatment concentrations (see Additional file 1; Supplemental figure 1).

**Figure 1:**
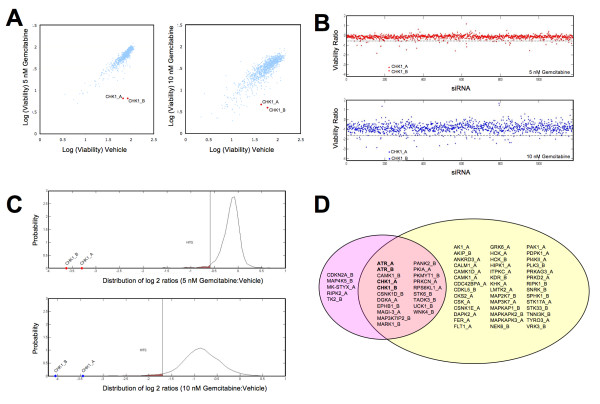
**HT-RNAi kinase screening for identification of sensitizers to gemcitabine**. HT-RNAi screens were performed on MIA PaCa-2 cells transfected with a siRNA library targeting 572 kinases followed by treatment with either vehicle or 5 nM or 10 nM gemcitabine. Cell viability was assessed and normalized to control wells. **(A) **Scatterplot of the log_2 _values of cell viability for gemcitabine plus siRNA treated cells versus vehicle plus siRNA treated cells showed CHK1 as a significant hit. **(B) **Plot of log_2 _ratios of gemcitabine/vehicle for each siRNA treated with either 5 nM or 10 nM gemcitabine. **(C) **Empirical Probability Distribution of log_2 _ratios of gemcitabine/vehicle (5 nM and 10 nM). Hit areas are highlighted in red. **(D) **Venn diagram of gene hits from both the 5 nM (highlighted in pink) and 10 nM (highlighted in yellow) gemcitabine synthetic lethal RNAi screen.

The HT-RNAi screen involved transfecting MIA PaCa-2 pancreatic cancer cells with validated siRNA library targeting 572 kinases followed by treatment at 24 hours with either vehicle or low concentration (5 or 10 nM) gemcitabine and with further incubation for an additional 72 hours. Cell viability was assessed using a luminescence-based cell number assay and the data was analyzed as described in Materials and Methods. Two independent HT-RNAi screens were conducted using 5 and 10 nM gemcitabine (Figure 1). The raw cell viability data was normalized to untreated wells within each assay plate. Synthetic lethal RNAi screening results are shown as a scatterplot of the log_2 _values of normalized cell viability for siRNA plus gemcitabine treated cells versus siRNA plus vehicle treated cells (Figure 1A). Results identified CHK1 as a significant hit. Log_2 _viability ratios of individual siRNA for the kinase siRNA screen were calculated (see Additional file 2).

**Figure 2:**
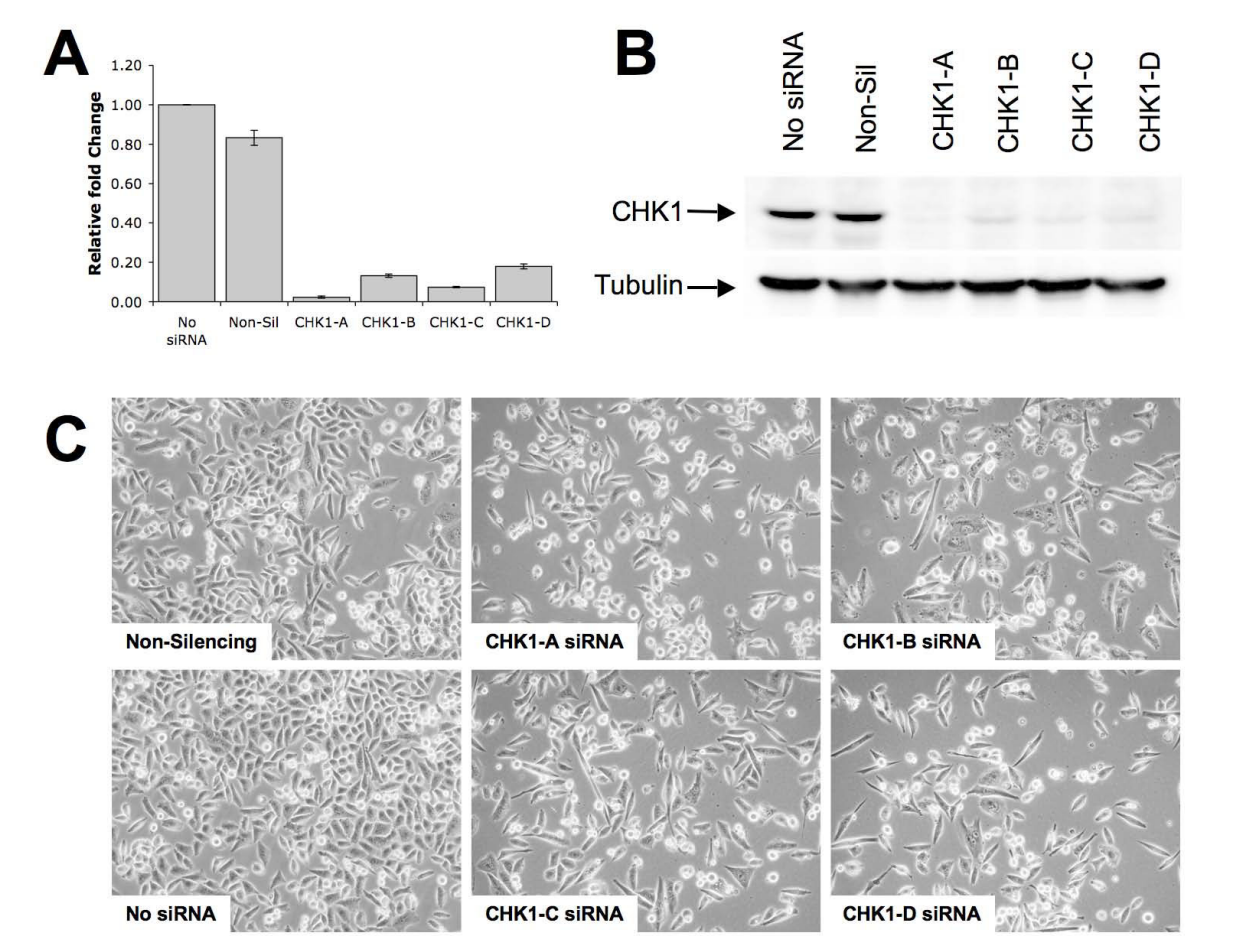
**Validation of gene silencing by CHK1 siRNA**. MIA PaCa-2 cells were transfected with either CHK1 or control siRNA and allowed to grow for 48–72 hrs. **(A) **Total RNA from the siRNA treated MIA PaCa-2 cells was isolated at 48 hrs and analyzed by qRT-PCR for CHK1 expression. CHK1 expression for each siRNA treatment was compared to untreated cells. GAPDH was used as an internal control for all the samples and fold change was calculated by normalizing all the data to GAPDH expression. **(B) **Lysates from CHK1 siRNA treated MIA PaCa-2 cells were prepared at 72 hrs post transfection and analyzed by western blot for expression of CHK1 protein using an anti-CHK1 antibody. **(C) **CHK1 siRNA treated cells showed decreased growth of MIA PaCa-2 cells at 72 hours after siRNA transfection when compared to no siRNA treatment or non-silencing siRNA treatment. Cell images were taken at 20× magnification.

Further visualization of the screening data included dot plots of log_2 _viability ratios of (siRNA + gemcitabine)/(siRNA + vehicle) for both the 5 nM and 10 nM concentrations (Figure 2B) and Empirical Probability Distribution of the log_2 _ratios for the 5 nM and 10 nM concentrations (Figure 1C). Both analyses showed that CHK1 siRNA highly potentiated gemcitabine response. Significant siRNA hits from both the screens are shown in the Venn diagram (Figure 1D). The results idenified 25 siRNA that potentiated the effect of 5 nM gemcitabine and 62 siRNA that were potentiators at 10 nM gemcitabine. Of interest was the finding that 20 siRNA were common on both lists. These overlapping hits included both siRNA targeting CHK1 as well as both siRNA targeting ATR. Several other interesting candidate genes were also identified such as CAMK1, STK6, PANK2 and EPHB1, all of which have been previously reported as being involved in cancer (Figure 1D) [22-25].

### Validation of gene silencing by CHK1 siRNA

To demonstrate the silencing efficiency of the siRNA targeting CHK1 or CHK2, MIA PaCa-2 cells were transfected with four CHK1 or CHK2 siRNA targeting different sequences or non-silencing siRNA. The experiment was run in duplicate and cells were incubated at 37°C for 48 hours prior to RNA extraction or 72 hours prior to the preparation of protein lysates. Expression analysis using qRT-PCR clearly showed that CHK1 (Figure 2A) and CHK2 (see Additional file 1; Supplemental figure 2) genes were silenced by all the four siRNA used, respectively. For all the qRT-PCR experiments, GAPDH was used as the internal control. In addition, cell lysates were analyzed by western blot using an anti-CHK1 antibody (Figure 2B) and images of the siRNA treated cells were captured (Figure 2C). Results show that all four CHK1 siRNA were able to reduce the CHK1 mRNA and protein levels compared to non-silencing control siRNA. The Western blots were also probed with anti-Tubulin antibodies to demonstrate equal protein loading (Figure 2B). MIA PaCa-2 cells treated with CHK1 siRNA showed decreased growth compared to non-silencing siRNA treated cells and no siRNA control (Figure 2C).

### Gene silencing of CHK1 potentiates the response to gemcitabine

**Figure 3:**
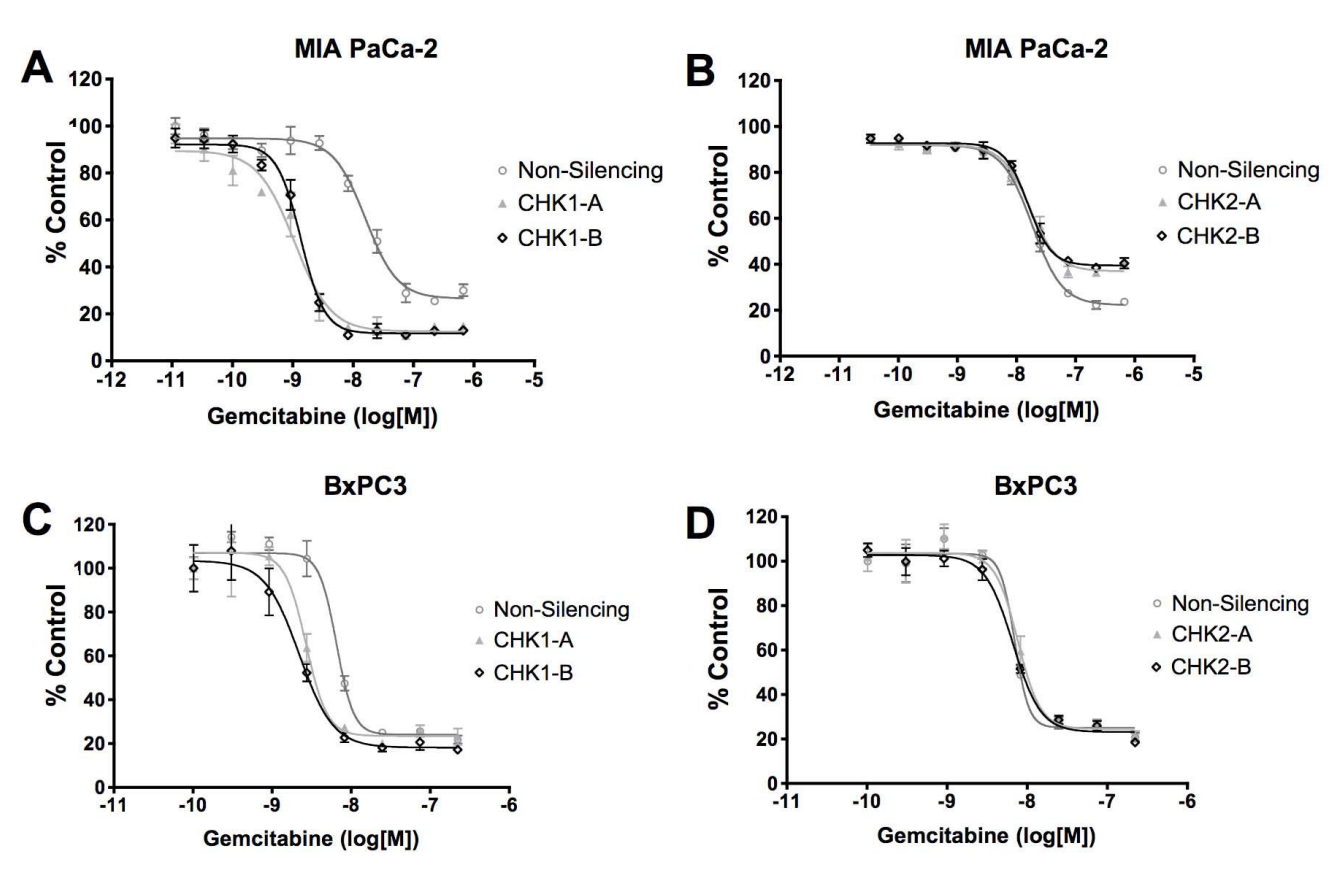
**Validation of CHK1 as a sensitizing target to gemcitabine in pancreatic cancer cells**. MIA PaCa-2 and BxPC3 pancreatic cancer cells were transfected with either CHK1, CHK2 or non-silencing siRNA. After 24 hours, cells were treated with varying concentrations of gemcitabine and incubated for an additional 72 hours. Cell number was assessed and data was normalized to siRNA plus vehicle control and plotted. Silencing of CHK1 showed potentiation of gemcitabine response in **(A) **MIA PaCa-2 and **(C) **BxPC3 cells as seen by the shift in the dose response curves. Silencing of CHK2 did not affect the response to gemcitabine in either **(B) **MIA PaCa-2 cells or **(D) **BxPC3 cells. Data is representative of three independent experiments.

In order to validate the synthetic lethal screening result indicating CHK1 as a sensitizing target for improving gemcitabine response, we generated drug dose response curves of MIA PaCa-2 cells treated with gemcitabine in the presence of CHK1, CHK2 and non-silencing siRNA (Figure 3). Interestingly, silencing of CHK1 potentiates the anti-proliferative effect of gemcitabine as seen by the shift in the dose response curves. The IC_50 _of CHK1 siRNA A and B plus gemcitabine treatment were 1.05 +/- 0.19 nM and 1.35 +/- 0.15 nM, respectively compared to an IC_50 _value of 15.8 +/- 1.2 nM for non-silencing control siRNA. Similar effects were seen with the CHK1 C & D sequences (data not shown). Furthermore, we used CHK2 siRNA A & B for comparison showing minimal change in IC_50 _values (Figure 3B). Similar effects were seen with the CHK2 C & D sequences (data not shown). We next validated the sensitization results in another human pancreatic cancer cell line, BxPC3. Drug response IC_50 _values in BxPC3 cells showed similar decrease from 6.9 +/- 2.4 nM for non-silencing to 2.8 +/- 0.4 nM and 2.4 +/- 0.6 nM for CHK1-A and CHK1-B siRNA respectively (Figure 3C). This effect was notably absent in the CHK2 siRNA-treated cells (Figure 3B and 3D).

### Real-time kinetic analysis of gemcitabine sensitization in pancreatic cells

**Figure 4:**
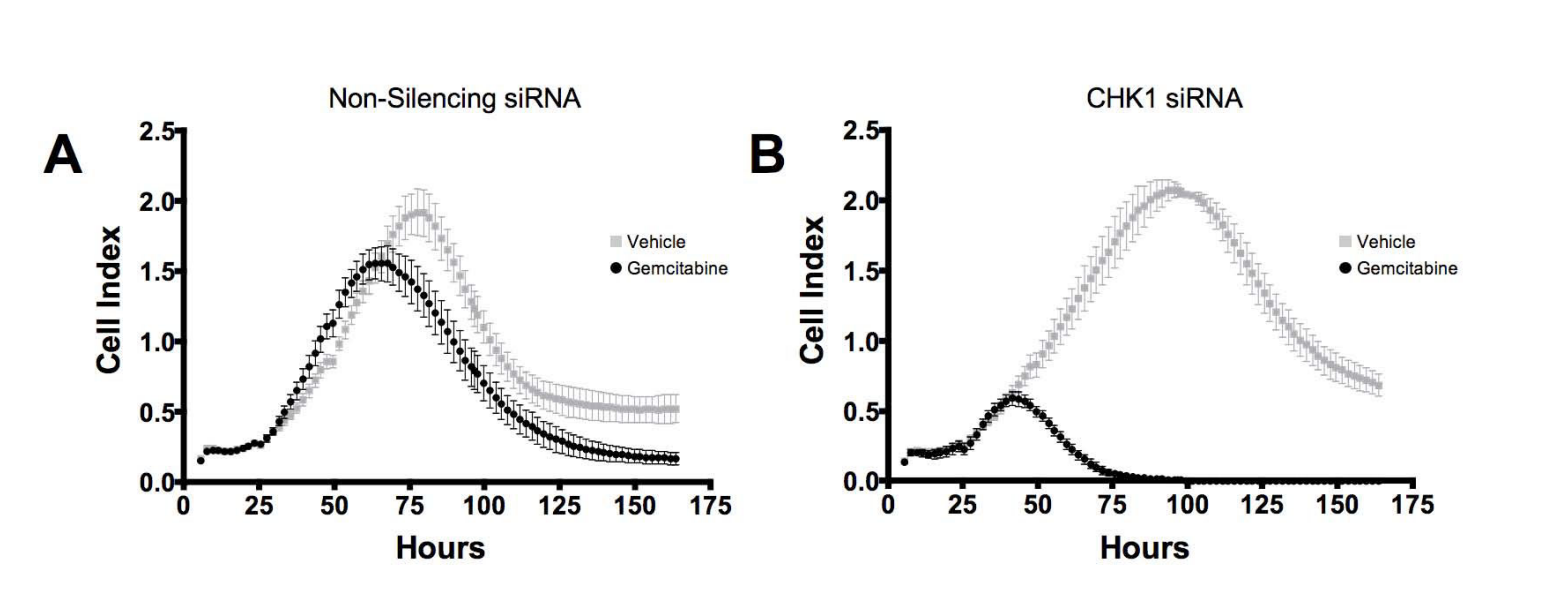
**Kinetic analysis of CHK1 siRNA induced sensitization of gemcitabine response**. MIA PaCa-2 cells were transfected with either CHK1 siRNA or non-silencing siRNA and at 24 hours post transfection, cells were treated with either vehicle or 10 nM gemcitabine. Growth was assessed by impedance measurements at 1-hour intervals and cell index was plotted as a function of time. **(A) **Treatment of cells with non-silencing siRNA and either vehicle or gemcitabine showed a slight decrease in cell growth by gemcitabine. **(B) **Pretreatment with CHK1 siRNA caused a pronounced decrease in cell growth in the gemcitabine treated cells compared to the vehicle treated cells. Data is representative of three independent experiments.

We next examined the effect of CHK1 siRNA and gemcitabine treatment on pancreatic cancer cells using label-free impedance growth assays (Figure 4). The impedance analysis showed that treatment of MIA PaCa-2 cells with non-silencing siRNA plus 10 nM gemcitabine showed slight decrease in cell number compared to non-silencing siRNA plus vehicle treatment (Figure 4A). Treatment of MIA PaCa-2 cells with CHK1-A siRNA and 10 nM gemcitabine showed a very potent reduction in cell growth compared to CHK1-A siRNA plus vehicle treatment (Figure 4B). Similar results were seen with other CHK1 siRNA (Data not shown). These results further demonstrate the potentiation of gemcitabine activity by CHK1 silencing.

### CHK1 inhibitors sensitize pancreatic cancer cells to gemcitabine

**Figure 5:**
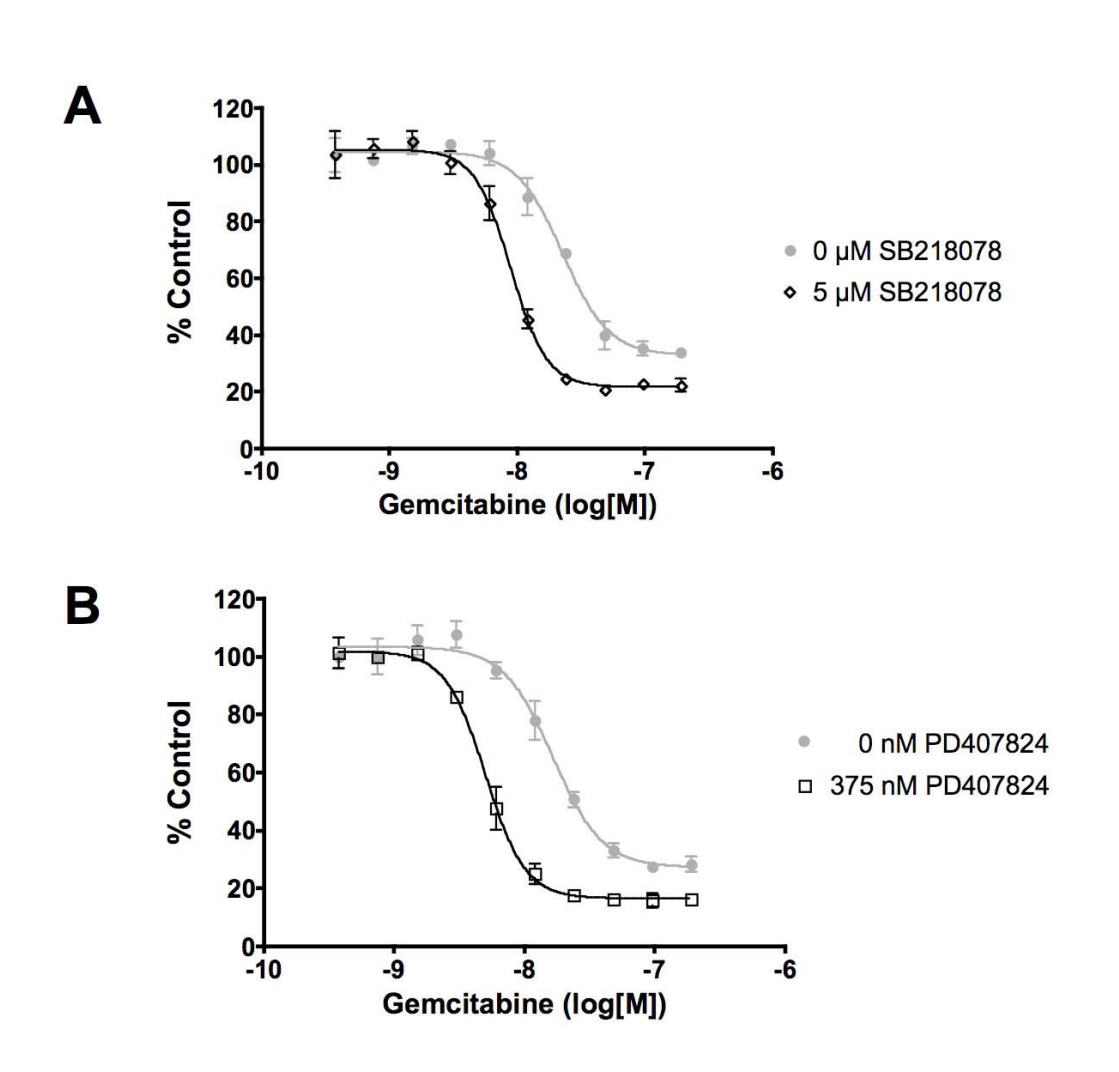
**CHK1 inhibitors potentiate gemcitabine response**. Treatment of MIA PaCa-2 cells with the CHK1 inhibitors **(A) **SB 218078 or **(B) **PD 407824 in combination with varying concentrations of gemcitabine resulted in a shift of the dose response curves suggesting potentiation of the gemcitabine response. Cell number was assessed and data was normalized to vehicle control and plotted. Data is representative of three independent experiments.

To confirm CHK1 as a sensitizing target for gemcitabine, we treated MIA PaCa-2 pancreatic cancer cells with CHK1 inhibitors SB 218078 and PD 407824 (Figure 5A &5B). MIA PaCa-2 cells treated with 5 μM SB 218078 followed by varying concentrations of gemcitabine resulted in a shift of the dose response curve and decreased the IC_50 _values from 22.5 +/- 2.0 nM for vehicle treatment to 8.8 +/-0.6 nM for SB 218078 treatment (Figure 5A). Similarly, MIA PaCa-2 cells treated with 375 nM PD 407824 and gemcitabine resulted in a shift of the dose response curve and a decrease of the IC_50 _values from 17.5 +/- 1.8 nM for vehicle treatment to 5.0 +/- 0.4 nM for PD 407824 treatment (Figure 5B).

## Discussion

In this study, we utilized a synthetic lethal screen based on high throughput RNAi to identify functionally relevant genes that could potentiate the response of pancreatic cancer cells to gemcitabine, the standard agent in pancreatic cancer chemotherapy. Literature review shows that combination therapies involving gemcitabine and other agents, such as axitinib, cisplatin, and fluorouracil are currently being studied [26-28]. Our approach to identifying combination partners for gemcitabine involves the application of a HT-RNAi functional genomics platform. Kinases are often considered to be prime drug targets because they are involved in numerous cellular pathways and are often deregulated in cancer cells. Therefore, we utilized a kinome-based HT-RNAi screening methodology to identify genes that sensitize pancreatic cancer cells to the cytotoxic effects of gemcitabine. The siRNA library used targets 572 kinases with two validated sequences per gene. Screening results identified at least 18 genes as potential sensitizing targets for two different concentrations of gemcitabine (Figure 1D). Several of these gene targets such as STK6 [29,30] and ATR [31] have previously been studied as therapeutic targets in pancreatic cancer. Another target, CAMK1 has been identified as being anti-apoptotic, and a report by Franklin *et al. *suggested that ROI-generating treatments trigger the activation of the calcium/calmodulin-dependent kinases (CaM-kinases), which in turn have a role in preventing apoptosis [32]. ATR, CHK1 and PKMYT1 are involved in DNA damage and G2/M cell cycle checkpoint, which clearly justifies them as good sensitizers of gemcitabine therapy [31,33,34]. Notably, the CHK1 kinase emerged as one of the most significant targets for gemcitabine sensitization and was followed up for further studies. Validation of gene silencing was performed by qRT-PCR and western blot analysis using four siRNA sequences targeting CHK1, two of which were used in the HT-RNAi screen (Figure 2A–B). Furthermore, treatment of MIA PaCa-2 cells with CHK1 siRNA resulted in decreased cell proliferation when compared to non-silencing control (Figure 2C), which is consistent with previous observations that silencing of CHK1 results in increased S and G2/M arrest [35]. Preliminary analysis of CHK1 siRNA in our studies also showed S and G2/M arrest (data not shown). It is worth noting that we performed HT-RNAi screening in one pancreatic cancer cell line and this might reflect the biological behavior of clinical pancreatic cancer only to a limited degree. Further validation of our results will need to be done in other pancreatic cancer cell lines.

**Figure 6:**
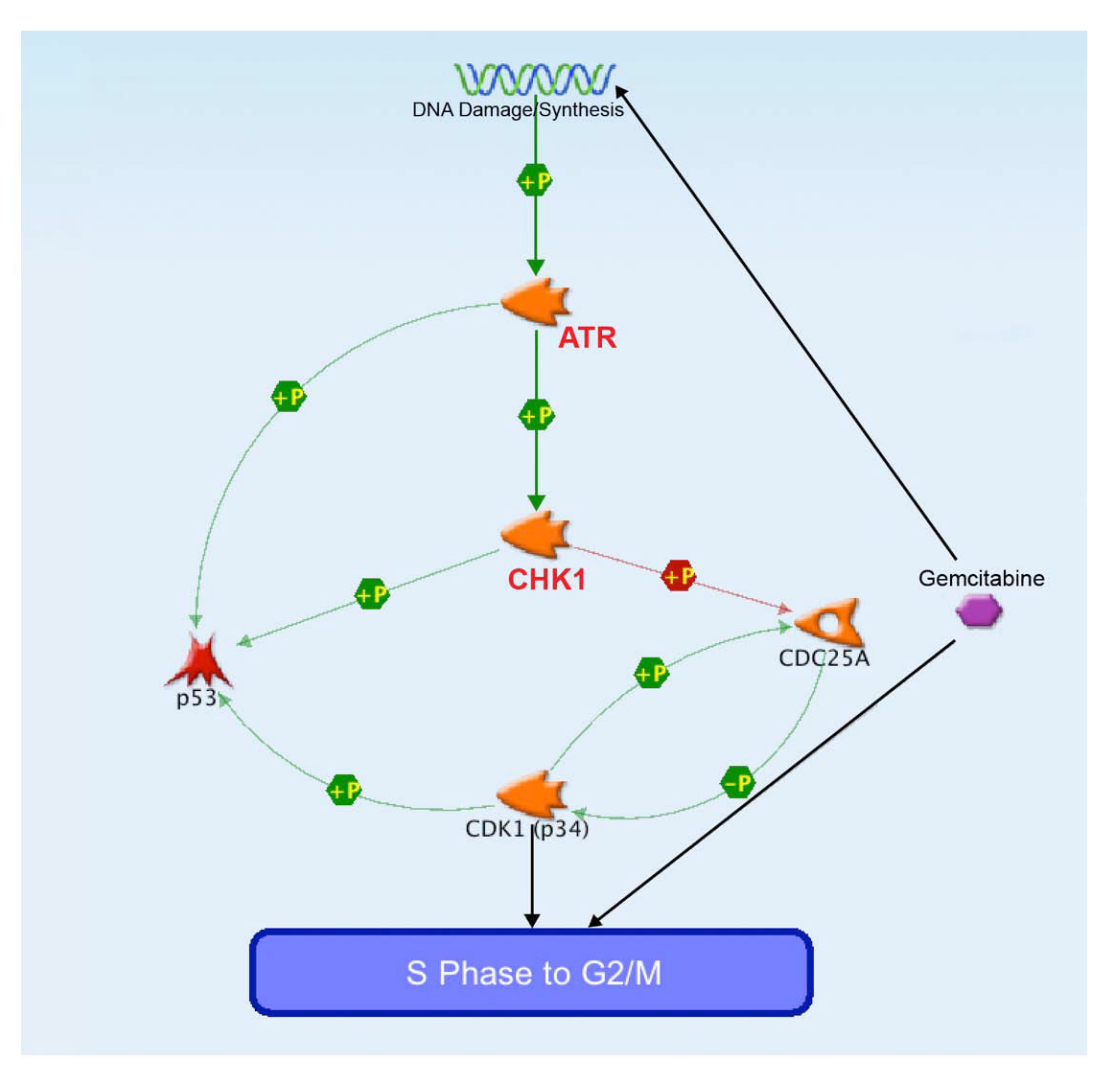
**Schematic of the role of CHK1 and ATR in sensitization to gemcitabine**. Genes identified as synergistic to gemcitabine in the RNAi kinase screens are shown in red. Gemcitabine induced DNA damage results in the phosphorylation and activation of serine/threonine-protein kinase CHK1 by ATR. The activated CHK1 then phosphorylates Cdc25A, leading to cell cycle arrest in G2/M. This rapid response via CHK – Cdc25A pathways additionally is followed by the p53-mediated maintenance of G1/S arrest. Tumor suppressor p53 plays a key role in the G2/M checkpoint arrest as well. In the maintenance stage, ATR phosphorylates Ser15 of p53 directly and Ser20 through activation of CHK1. Phosphorylated p53 activates its target genes, including cyclin-dependent kinase inhibitor 1A (p21), which binds to cyclin-dependent kinase 2 (Cdk2) and cyclin-dependent kinase 4 (Cdk4). Map was constructed with MapEditor (GeneGO).

CHK1 is a protein kinase that plays a key role in the DNA damage checkpoint signal transduction pathway (Figure 6) [33,36]. In mammalian cells, CHK1 is activated in response to chemotherapeutic agents that disrupt or block DNA replication such as hydroxyurea, pemetrexed, and gemcitabine, as well as ionizing and ultraviolet radiation [37-40]. Activation of CHK1 in dividing cells normally induces an arrest in the cell cycle to allow for DNA repair and completion of replication prior to mitosis. It is postulated that inhibition of CHK1 results in the release of cells from checkpoint arrest, allowing progression into mitosis with unreplicated or damaged DNA, which can ultimately cause apoptosis [41,42]. This results in increased sensitization of cells to DNA damaging agents such as gemcitabine. Here we utilize CHK1 inhibitors as a means to abrogate cell cycle arrest and prevent DNA repair following treatment with gemcitabine. A recent study by Parsels *et al. *has shown that PD-321852 inhibited CHK1 in MIA PaCa-2 cells as evidenced by stabilization of Cdc25A and a synergistic loss of CHK1 protein was observed in combination with gemcitabine [43]. In these cells, the results fit the prevailing model: inhibition of CHK1 led to abrogation of gemcitabine-induced Cdc25A degradation, premature mitotic entry, and sensitization to gemcitabine. Therefore, in MIA PaCa-2 cells, CHK1 is involved in destabilization of Cdc25A, via phosphorylation by CHK1 at multiple sites, which in turn results in inactivation of cyclin-dependent kinase 1 complexes and G2 arrest and/or inactivation of cyclin-dependent kinase 2 complexes and intra-S-phase arrest [43].

In order to validate the functional association of CHK1 silencing with gemcitabine treatment, we treated pancreatic cancer cells with CHK1 siRNA followed by treatment with gemcitabine. Results indicate that CHK1 silencing shifted the EC_50 _of gemcitabine approximately ten-fold in MIA PaCa-2 cells (Figure 3A) and approximately three-fold in BxPC3 cells (Figure 3C). This effect was notably absent in the CHK2 siRNA-treated cells (Figure 3B and 3D). The CHK1/CHK2 proteins potentiate separate signal transduction pathways, both of which play a role in cell cycle arrest in response to DNA damage [33]. However, our data suggest that CHK1 is essential for maintaining gemcitabine-induced S-phase arrest whereas CHK2 is not. This is in accordance with previously published data [39,40].

Loss-of-function screening using siRNA libraries has previously been used to identify genes that modulate gemcitabine activity in cervical and pancreatic cancer cell lines [12,44]. Using a screen of pooled siRNA targeting ~20,000 genes, Bartz *et al. *identified CHK1 as one of several genes that shifted the IC_50 _of gemcitabine treatment greater than two-fold in HeLa cervical cancer cells [12]. Using pancreatic cancer cell lines, Giroux *et al*. screened an siRNA library targeting kinases and found that CHK1 silencing increased apoptosis by 2.1 fold [44]. Interestingly, six of our top eighteen significant genes were also identified by Giroux *et al. *as significant "hits." These genes include ATR, DGKA, KDR, RIPK1, CHK1 and MAPKAP1. Our screen not only identified CHK1 as a gemcitabine sensitizer, but also showed that CHK1 siRNA had the highest degree of potentiation of gemcitabine activity.

CHK1 targeting has recently become a focus for pharmaceutical companies [41,45]. CBP501, a G2 checkpoint abrogator with activity against CHK1 is currently undergoing clinical development [46]. Other CHK1 inhibitors undergoing clinical development include XL844 [47], AZD7762 [48], and 5,10-dihydro-dibenzo [b, e] [1, 4]diazepin-11-one [49]. In the past, nonselective CHK1 inhibitors like UCN-01 and 17-AAG have been well tolerated in Phase I clinical trials [50-52]. The data presented here suggests that administering these CHK1 inhibitors in combination with gemcitabine would be more effective in treating pancreatic cancer patients than gemcitabine alone. Moreover, *in vivo *experiments demonstrating that inhibitors of CHK1 can increase the anti-tumor activity of gemcitabine have already been conducted in colorectal [53] and pancreatic cancer xenografts [47].

## Conclusion

This study utilized a synthetic lethal RNAi screen targeting 572 different kinases to identify sensitizing targets to gemcitabine in pancreatic cancer cells. The RNAi screening identified several genes as potential sensitizing targets, but showed that CHK1 had the best sensitizing activity. We demonstrated potentiation of gemcitabine activity by showing a shift in the dose response curve of gemcitabine by CHK1 siRNA. In addition, we functionally-validated the combination of gemcitabine and CHK1 inhibitors as a potential treatment for pancreatic cancer patients. The preclinical finding of inhibition of CHK1 as a sensitizing target for gemcitabine is currently being tested in clinical trials. Collectively, the data presented here clearly show that synthetic lethal, high throughput RNAi screening is a powerful and robust platform for screening hundreds or thousands of genes for the identification of novel interacting targets that can enhance the activity of existing chemotherapeutic agents. This high throughput RNAi screening platform would provide an expedited method for determining effective combination therapies.

## Competing interests

The authors declare that they have no competing interests.

## Authors' contributions

DOA, SM, JMT and DDV were responsible for the initial conception and design of this study. DOA was responsible for planning of the experiments. RNAi screening was performed by IMG and MCH and analyzed by SA, JAK and AC. Functional validation of siRNA sensitization and drug synergy was performed by IMG. KMB, GDB and SA performed the validation of gene silencing. DOA, GDB, SA, and MCH were involved in the writing of the manuscript. All authors have read and approved the final version.

## Supplementary Material

Additional file 1 **Supplemental Figures**. The data provided represents the dose response of MIA PaCa-2 cells to gemcitabine (supplemental figure 1) and the validation of CHK2 gene silencing in MIA PaCa-2 cells by qRT-PCR (supplemental figure 2). Click here for file

Additional file 2 **HT-RNAi screening log_2 _ratios**. The data provided shows the log_2 _ratios of normalized viability of siRNA plus gemcitabine-treated MIA PaCa-2 cells versus siRNA plus vehicle treated MIA PaCa-2 cells. Click here for file
