# Supplementary material for: Synthetic lethal RNAi screening identifies sensitizing targets for gemcitabine therapy in pancreatic cancer
Source: J Transl Med. 2009 Jun 11;7:43. doi: 10.1186/1479-5876-7-43 (PMC2702280; doi:10.1186/1479-5876-7-43)
Supplement: Additional file 1 — Supplemental Figures. The data provided represents the dose response of MIA PaCa-2 cells to gemcitabine (supplemental figure 1) and the validation of CHK2 gene silencing in MIA PaCa-2 cells by qRT-PCR (supplemental figure 2). [file 1479-5876-7-43-S1.doc]

**Additional file 1: Supplemental figures**

**Supplemental figure 1:**


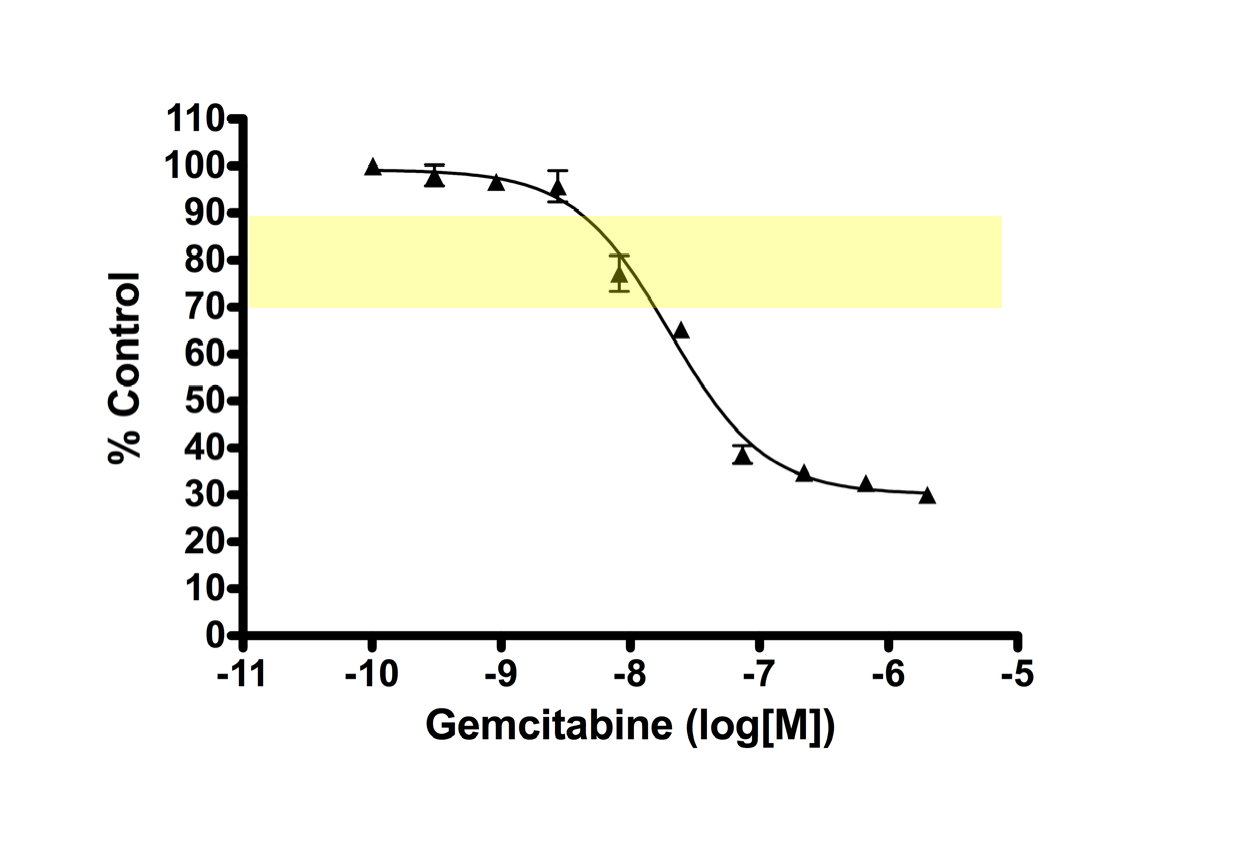


**Supplemental figure 1. Dose response of MIA PaCa-2 pancreatic cancer cells to gemcitabine to calculate EC_10-30_ concentration.** MIA PaCa-2 cells were plated in 384-well plates and treated with varying concentrations of gemcitabine at 24 hr. Cells were incubated for 72 hr in the presence of drug and final cell number was determined using Cell Titer Glo. The targeted range between EC_10_ and EC_30_ is highlighted. The EC_10_ and EC_30_ concentrations were 3.06 nM and 9.75 nM respectively as determined using GraphPad Prism.

**Supplemental figure 2:**


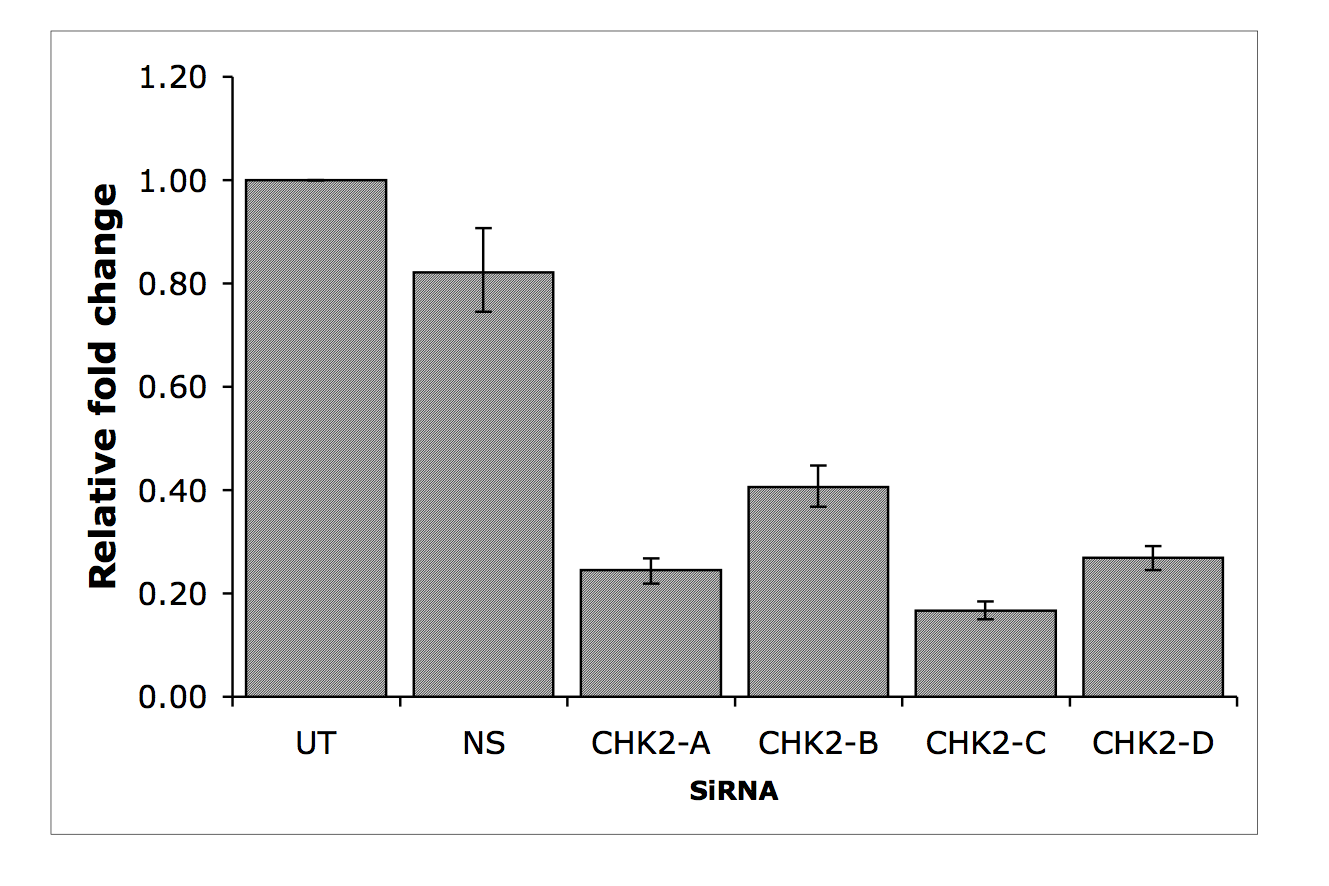


**Supplemental figure 2. Validation of CHK2 silencing.** Total RNA from the siRNA treated MIA PaCa-2 cells was isolated and analyzed by Q-RTPCR analysis for CHK2 expression. CHK2 expression for each CHK2 siRNA condition and non-silencing siRNA (NS) were normalized to untreated cells (UT). GAPDH was used as an internal control for all the samples and the fold change was calculated by normalizing all the data to GAPDH expression.
